# Unlocking reproducible transcriptomic signatures for acute myeloid leukaemia: Integration, classification and drug repurposing

**DOI:** 10.1111/jcmm.70085

**Published:** 2024-09-12

**Authors:** Haoran Chen, Jinqi Lu, Zining Wang, Shengnan Wu, Shengxiao Zhang, Jie Geng, Chuandong Hou, Peifeng He, Xuechun Lu

**Affiliations:** ^1^ School of Biomedical Engineering and Informatics Nanjing Medical University Nanjing China; ^2^ School of Management Shanxi Medical University Taiyuan China; ^3^ Department of Nephrology First Medical Center of Chinese PLA General Hospital, Nephrology Institute of the Chinese People's Liberation Army, National Key Laboratory of Kidney Diseases, National Clinical Research Center for Kidney Diseases, Beijing Key Laboratory of Kidney Disease Research Beijing China; ^4^ Department of Computer Science Boston University Boston Massachusetts USA; ^5^ Department of Hematology The Second Medical Center of Chinese PLA General Hospital, National Clinical Research Center for Geriatric Disease Beijing China; ^6^ Medical School of Chinese PLA Beijing China; ^7^ Department of Rheumatology and Immunology The Second Hospital of Shanxi Medical University Taiyuan China; ^8^ Key Laboratory of Coal Environmental Pathogenicity and Prevention at Shanxi Medical University, Ministry of Education Taiyuan Shanxi China; ^9^ Basic Medicine College, Shanxi Medical University Taiyuan China; ^10^ Shanxi Key Laboratory of Big Data for Clinical Decision Shanxi Medical University Taiyuan China

**Keywords:** acute myeloid leukaemia, CSAMLdb database, machine learning, transcriptomic analysis

## Abstract

Acute myeloid leukaemia (AML) is a highly heterogeneous disease, which lead to various findings in transcriptomic research. This study addresses these challenges by integrating 34 datasets, including 26 control groups, 6 prognostic datasets and 2 single‐cell RNA sequencing (scRNA‐seq) datasets to identify 10,000 AML‐related genes (ARGs). We focused on genes with low variability and high consistency and successfully discovered 191 AML signatures (ASs). Leveraging machine learning techniques, specifically the XGBoost model and our custom framework, we classified AML subtypes with both scRNA‐seq and bulk RNA‐seq data, complementing the ELN2022 classification approach. Our research also identified promising treatments for AML through drug repurposing, with solasonine showing potential efficacy for high‐risk AML patients, supported by molecular docking and transcriptomic analyses. To enhance reproducibility and customizability, we developed CSAMLdb, a user‐friendly database platform. It facilitates the reuse and personalized analysis of nearly all results obtained in this research, including single‐gene prognostics, multi‐gene scoring, enrichment analysis, machine learning risk assessment, drug repositioning analysis and literature abstract named entity recognition. CSAMLdb is available at http://www.csamldb.com.

## 1 INTRODUCTION

Acute myeloid leukaemia (AML) is a haematopoietic malignancy with an incidence of 4.1 per 100,000 individuals and a mortality rate of 2.7 per 100,000 annually. The complexity and evolving nature of AML makes standardizing treatment challenging. RNA‐sequencing, now emerging in clinical diagnostics, provides deeper biological insight and improves disease classification, crucial for personalized therapy. However, its full clinical integration faces challenges due to diagnostic and identification issues for targeted treatments.^1^, ^2^, ^3^, ^4^

Transcriptomics has been extensively utilized for screening molecular biomarkers and diagnosing and treating diseases.^5^ Previous studies have compared the transcriptomes of AML and normal samples,^6^, ^7^, ^8^, ^9^, ^10^, ^11^, ^12^, ^13^, ^14^ proposing numerous AML‐related mechanisms, including thrombocytopenia,^6^ haematopoietic damage,^7^ senescence,^8^ DNA methylation variation,^9^ dysregulation of proteoglycans, adhesion molecules, cytokines,^10^ disruption of the adipocytic niche,^11^ adipogenic shift,^12^ cytokine storm^13^ and mesenchymal stromal cell dysfunction.^14^ Despite these findings, challenges remain, such as the absence of batch‐matched normal controls and cross‐validated AML transcriptomic data. The relevance of these factors to AML diagnosis and prognosis, as well as the occurrence of multiple pathogenic mechanisms, remains unclear.

This study integrates 34 datasets using a standardized pipeline to identify consistent, low‐variability AML signatures (ASs). We aimed to address the heterogeneity and batch effects in various AML studies by leveraging these signatures. Our approach includes an advanced machine‐learning framework to construct robust risk prediction and cell classification models for AML patients. Additionally, we assessed solasonine as a potential inhibitor for high‐risk AML cases. Finally, we developed CSAMLdb, a user‐friendly database for replicable verification and personalized analysis of our findings.

## 2 MATERIALS AND METHODS

### 2.1 Data preprocessing and functional annotation

The initial data were sourced from the GEO (https://www.ncbi.nlm.nih.gov/geo/) and TCGA (https://portal.gdc.cancer.gov/) databases. Data preprocessing and scRNA‐seq analysis were conducted using Seurat package, while differential expression analysis employed DESeq2 or limma. AML‐related genes (ARGs) were identified based on genes present in at least one dataset, using the gene mean consistency score (GMCS) and coefficient of variation (CV) to generate and validate ASs. Radial graphs assessed the similarity between various AML datasets and AS, determining a similarity score based on the proportion of overlapping genes. To elucidate the biological processes and key pathways related to AML, Gene Ontology (GO) and Kyoto Encyclopedia of Genes and Genomes (KEGG) pathway enrichment analysis were conducted. Drugs analysis targeting AS was performed using the DGIdb and DrugTargetEnrich databases, focusing on drug enrichment at the ATC 3 level. Detailed content can be found in the Supplementary Methods.

### 2.2 Integrating machine learning algorithms for prognostic signature discovery

Non‐negative matrix factorization (NMF) was applied to the TCGA‐beatAML dataset using strategically selected subsamples in iterations to identify intrinsic AML subtypes. The optimal cluster count was determined using the cumulative distribution function (CDF). An ensemble approach integrating 10 machine learning algorithms and 99 of their combinations was employed to identify prognostic markers. The methodology included:

- Screening mRNAs correlated with prognosis in the TCGA‐beatAML dataset via univariate Cox regression.
- Implementing the highlighted algorithms for leave‐one‐out cross‐validation (LOOCV) on the dataset to develop predictive frameworks.
- Testing these models on five distinct validation datasets (e.g. TCGA‐AML, GSE12417‐GPL570, etc.).
- Selecting the best model by evaluating Harrell's concordance index (C‐index) for each across all validation sets.
- Conducting a thorough evaluation by comparing the C‐index of our model against seven recognized AML prognostic models to determine predictive accuracy.^15^, ^16^, ^17^, ^18^, ^19^, ^20^, ^21^, ^22^

Details are provided in the Supplementary Methods.

### 2.3 Drug screening for high‐risk patients based on molecular docking

To identify potential therapeutic compounds for high‐risk patients, we conducted molecular docking assessments on 4454 compounds sourced from the PubChem database (https://pubchem.ncbi.nlm.nih.gov/). The target proteins for these assessments were derived from machine‐learning models, with their 3D structural models obtained from Uniprot (https://www.uniprot.org/), RCSB PDB (https://www.rcsb.org/), and AlphaFold (https://alphafold.ebi.ac.uk/) when necessary. We filtered these structural models, retaining only those determined via X‐ray diffraction with a resolution sharper than 2.50 Å.

The target proteins underwent preprocessing steps using AutoDockTools, including the removal of water molecules, addition of hydrogen atoms, computation of charges, and conversion to the pdbqt format suitable for docking procedures. Concurrently, small molecule drugs were prepared using Python's Pybel (version 0.15.5) and Openbabel (version 3.1.0) modules.

Following these preparations, molecular docking simulations were executed using qvina2, an enhanced version of AutoDock Vina. The docking results were analysed, and the ligand‐protein complexes were visualized using PyMOL (version 2.5.7). We focused on identifying drugs with a binding affinity of −5.0 kcal/mol or lower, indicating a favourable interaction between the target protein and the drug. From this subset, drugs that consistently ranked within the top 30 for binding affinity were prioritized.

### 2.4 XGBoost model for identifying cell origins in AML patients versus normal individuals

A machine‐learning model was developed using 191 AS genes to differentiate cells between AML patients and normal individuals. The scRNA‐seq datasets from GSE116256 served as the training set, with validation performed on GSE198052. Data preprocessing and dimensionality reduction via UMAP were conducted using the Seurat package. The XGBoost algorithm (v1.7.5.1) was implemented to create a gradient‐boosted decision tree model for binary classification, utilizing a logistic objective function. Model efficacy was assessed using the area under the curve (AUC). Key hyperparameters included a tree depth capped at 6, a learning rate (eta) set at 0.01, and column subsampling at a 0.5 rate, with the model trained over 1000 iterations.

To verify the model's accuracy in pinpointing AML patient cells, annotations were compared with entries in the ABC portal database for the GSE116256 dataset. Emphasis was placed on annotations from the corresponding publication and malignancy ratings established through inferCNV.^23^, ^24^, ^25^ For future cases where the origin of samples is undetermined, the XGBoost model can be initially used to identify cells from AML patients, followed by inferCNV deployment to identify malignant cells in the AML context, in the AML context.

### 2.5 Constructing a comprehensive signature for AML database

To enhance the reproducibility and applicability of our study for both researchers and clinicians, we developed a web‐based application using Shiny (v1.7.5) in R (v4.3.0). The underlying database architecture was designed with the DBI package (v1.1.3) to ensure streamlined data access and reuse. Our system is hosted on an Ubuntu 22.04 LTS platform.

### 2.6 Statistical analysis

All statistical analyses were performed in R (version 4.3.0). Univariate Cox regression analyses were conducted using the survival package (version 3.5–5), while the survminer package (version 0.4.9) was utilized for survival analysis and visualization. A *p*‐value threshold of <0.05 was set as the criterion for statistical significance.

## 3 RESULTS

### 3.1 Data preprocessing

**FIGURE 1:**
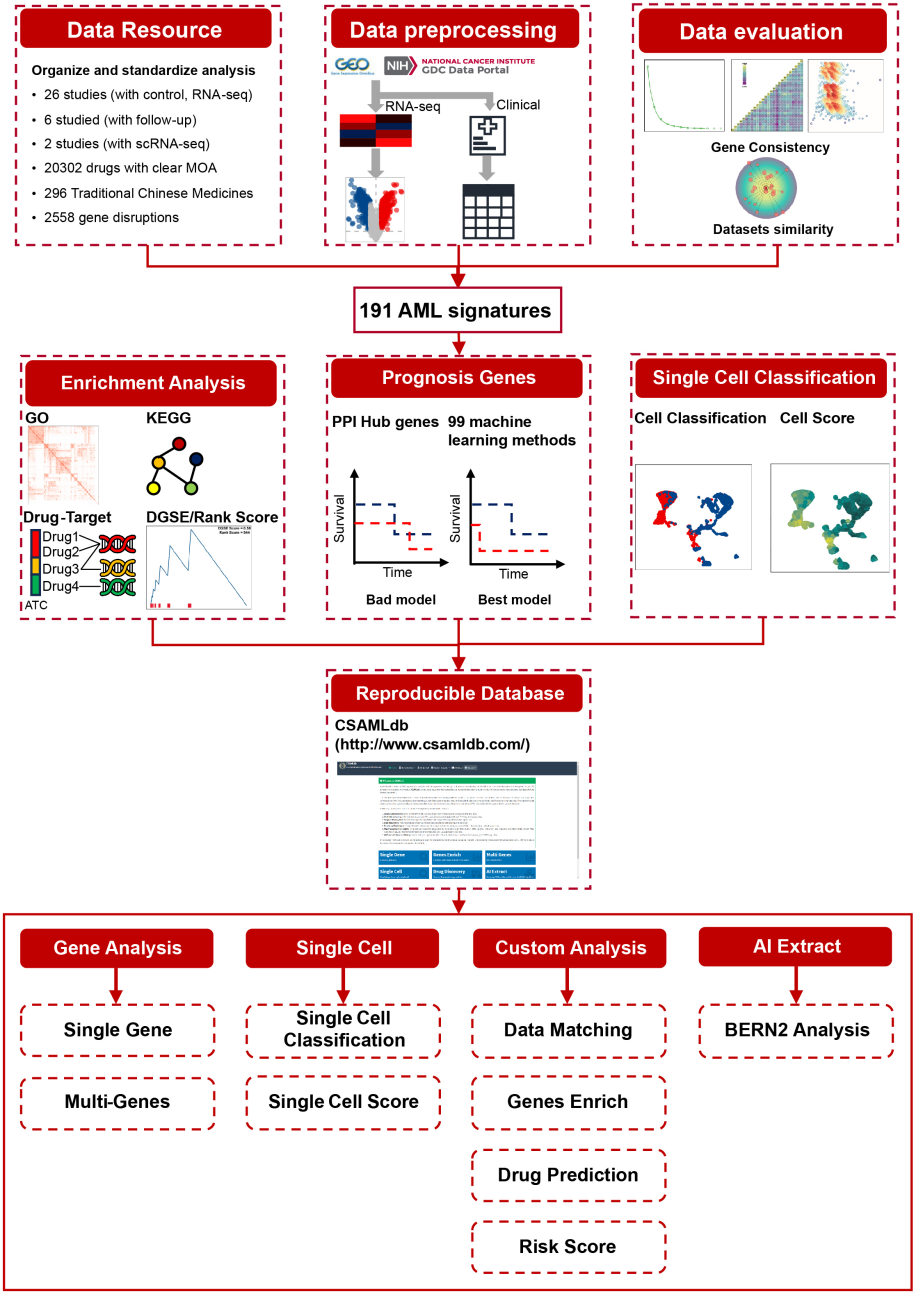
Research process flow chart. This flow chart outlines the sequential steps taken in the study, from initial data collection through to analysis and interpretation of results. BERN2, biomedical named entity recognition and normalization tool; CSAMLdb, comprehensive signature of acute myeloid leukaemia database; DGSE, disease gene signature enrichment; GO, Gene Ontology; KEGG, Kyoto encyclopedia of genes and genomes; scRNA‐seq, single‐cell RNA sequencing; PPI, protein–protein interaction.

The complete methodology and technical routes employed in this study are delineated in Figure 1. A total of 34 datasets fulfilling the predefined criteria were incorporated into the database (Table S1). Among them, 26 datasets had control groups, 6 included follow‐up data and 2 applied scRNA‐seq technology.

A consistent criterion was applied to identify differentially expressed genes (DEGs) across these 26 datasets, labelled AML1 through AML26. The resultant gene lists are shown in Figure S1A (Tables S2 and S3). The number of DEGs varied, with the highest being 1070 genes in AML12 and the lowest at 277 genes in AML6. Such variations in gene expression might arise due to differences in sample sizes, heterogeneity of bone marrow cell lines, distinct sequencing platforms, or inherent individual variances.^26^, ^27^

### 3.2 Evaluation of consistency among AML‐related genes

From the 26 datasets of DEGs, 10,000 ARGs were identified using a minimum occurrence of one as the selection criterion (Table S4). Despite the substantial total number of DEGs, subsequent analysis revealed that genes consistently recognized across multiple studies displayed limited reproducibility (Figure S1B). Notably, over 6000 genes were exclusive to just one study. Further examination of the overlap between any two studies and the proportion of genes replicated in only one study showed minimal intersection among some datasets (Figure S1C).

### 3.3 Determining AML signatures

Our initial analysis revealed limited reproducibility of most ARGs across various studies. To address this, we shifted our focus to genes with a higher frequency of occurrence in the dataset. The GMCS and CV for the ARGs were calculated using permutations from all 26 datasets (Figure S1D). Results indicated that ARGs appearing in ≥6 datasets had a higher GMCS and more stable CV. Based on this, genes present ≥6 times in the ARGs were identified as AML signatures, totaling 191 genes (Table S5). Further analysis revealed that all dataset captured the AML signatures entirely, with each exhibiting some level of overlap (Figure S1E).

### 3.4 Enrichment analysis of AML signatures

We employed both DGSE and RS algorithms to score the 26 individual AML datasets, revealing a diverse range of values. As detailed in the Supplementary Methods, each algorithm has its strengths in qualitative and quantitative assessments. Notably, a high score with one algorithm does not guarantee a similar score with the other, highlighting the distinct characteristics of each method. Specifically, RS emphasizes quantity, suggesting more AML‐related genes as its score increases, while DGSE leans towards qualitative evaluation, indicating a higher proportion of AML‐related genes as its score rises. A combined analysis aids in the initial appraisal of a dataset's relevance to AML (Figure S2A–C).

**FIGURE 2:**
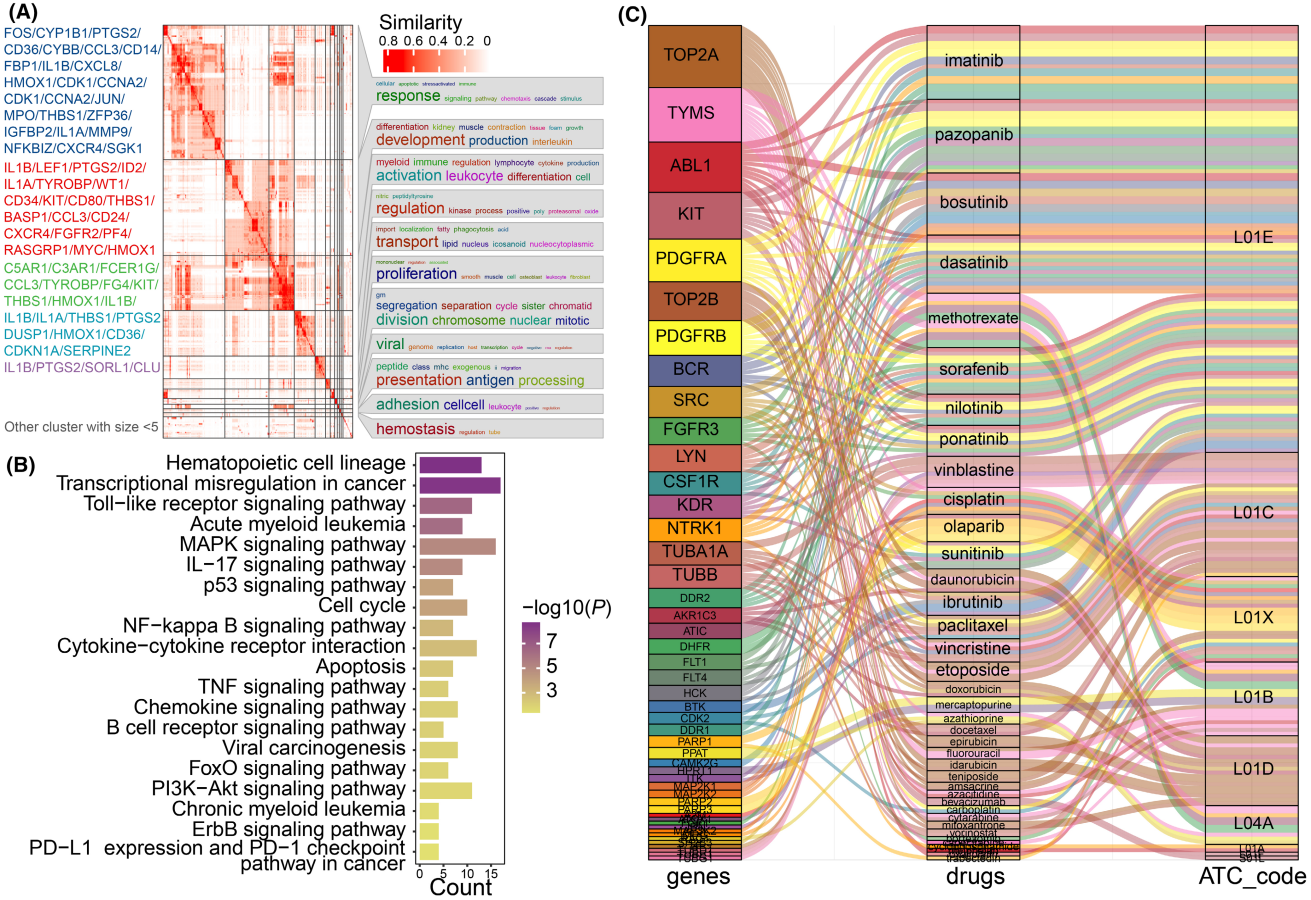
Functional characterization of AS through GO, KEGG, and drug–target interactions. (A) Similarity matrix heatmap of GO terms associated with AML. On the left side, key genes corresponding to the GO terms are listed in order of their frequency of occurrence. The heatmap is annotated on the right with word clouds that summarize the functional aspects of each GO term cluster. (B) KEGG pathway enrichment analysis of AS. **(C)** Sankey plot illustrating the relationships between genes, drugs, and their corresponding ATC Level 3 codes. The plot consists of three main components: genes (left), drugs (middle) and ATC codes (right). Each coloured line represents an interaction between a gene and a drug, indicating that the drug targets the specific gene. The colours of the lines are used to visually differentiate between different interactions for clarity. The size of each box (gene, drug, or ATC code) represents the number of connections it has. Detailed information about these interactions is provided in Table S6.

In the GO enrichment analysis for AS, 18 clusters were identified, with a particular emphasis on the top four due to their statistical significance and biological importance (Figure 2A). The primary cluster, marked by genes such as *FOS*, *CYP1B1* and *PTGS2*, was predominant in cell death and immune responses associated with AML.^28^ The second cluster, represented by genes *IL1B* and *LEF1*, underscored cellular differentiation and suggested stem cell‐like attributes in AML.^29^, ^30^ The third cluster, featuring pivotal genes *C5AR1* and *C3AR1*, highlighted immune regulation in AML. Meanwhile, the fourth cluster, encompassing *IL1B* and *THBS1*, highlighted oxidative stress in AML.^31^ Together, these findings elucidate key biological processes and pathways inherent in AML.

Diverse signalling pathways and cellular processes crucial to the pathogenesis and progression of AML were uncovered (Figure 2B). Overlaps with pathways observed in other cancers, including transcriptional mis‐regulation in cancer and chronic myeloid leukaemia, hint at a shared molecular underpinning across different tumours. Haematopoietic cell‐specific pathways, such as the haematopoietic cell lineage, were pinpointed, underlining AML's haematopoietic roots. Central regulatory pathways like p53, MAPK, TNF and PI3K‐Akt have vital roles in cell cycle regulation, apoptosis and immunity. These pathways interweave with networks, such as NF‐κB, FOXO and cytokine‐cytokine receptor interaction, emphasizing the intricate cellular signalling dynamics in AML.^32^ Immunomodulatory pathways like PD‐L1 expression, PD‐1 checkpoint and others were underscored as pivotal to AML's biology.^33^ Additionally, pathways like chemokine and ErbB signalling, crucial for cell migration and growth signals, were recognized. Collectively, these insights contribute to a comprehensive molecular picture of AML, revealing the complexity of its pathogenesis and highlighting potential therapeutic target.

To uncover pharmacological agents aligned with AML molecular signatures, an enrichment analysis was conducted using the DGIdb and DrugTargetEnrich databases, referencing ATC Level 3 codes (Figure 2C). Significant enrichment was observed in several anticancer drug classes, particularly antimetabolites (L01B), plant alkaloids and other natural products (L01C) and protein kinase inhibitors (L01E). These classes registered statistically significant adjusted *p*‐values below 0.001 and are supported by corresponding literature (Tables S6 and S7). This underscore the potential for targeted therapies to disrupt critical AML pathways and potentially improve patient outcomes. These drugs target pivotal AML‐related genes, such as TYMS and DHFR related to DNA/RNA metabolism, and tyrosine kinases represented by FLT1 and KIT. Although many of these drugs are already part of current AML treatment regimens, integrating a prognosis‐driven strategy remains crucial to pinpoint the most effective therapeutic avenues.

### 3.5 Prognostic analysis and identification of key AML signatures

Using STRING, a PPI network centred on AML signatures was established, which yield a significant enrichment *p*‐value of <1.0e^−16^. This network consisted of 191 nodes interconnected by 1217 edges. Through the CytoHubba plug‐in in Cytoscape and leveraging the MCC algorithm, we pinpointed 10 hub genes: *IL1B*, *CXCL8*, *IL1A*, *CXCR4*, *MMP9*, *PTGS2*, *CD36*, *JUN*, *ANXA5* and *MPO*. Survival analysis using the TCGA‐LAML and TCGA‐beatAML datasets revealed that only *JUN* and *MPO* were significant prognostic markers (*p* < 0.05) (Figures S3 and S4).

To better assess patient prognosis and perform prognostic stratification, we applied the NMF algorithm to 191 AML features. NMF clustering on the 213 AML samples in the TCGA‐beatAML dataset revealed that a factorization rank of 2 resulted in the most cohesive clusters (Figures S5A and 5B). Despite AML's evident heterogeneity, a binary classification at the transcriptomic level might offer clearer insights into key genes or pathways for AML prognosis.

### 3.6 Integrating machine learning algorithms for prognostic signature discovery

**FIGURE 3:**
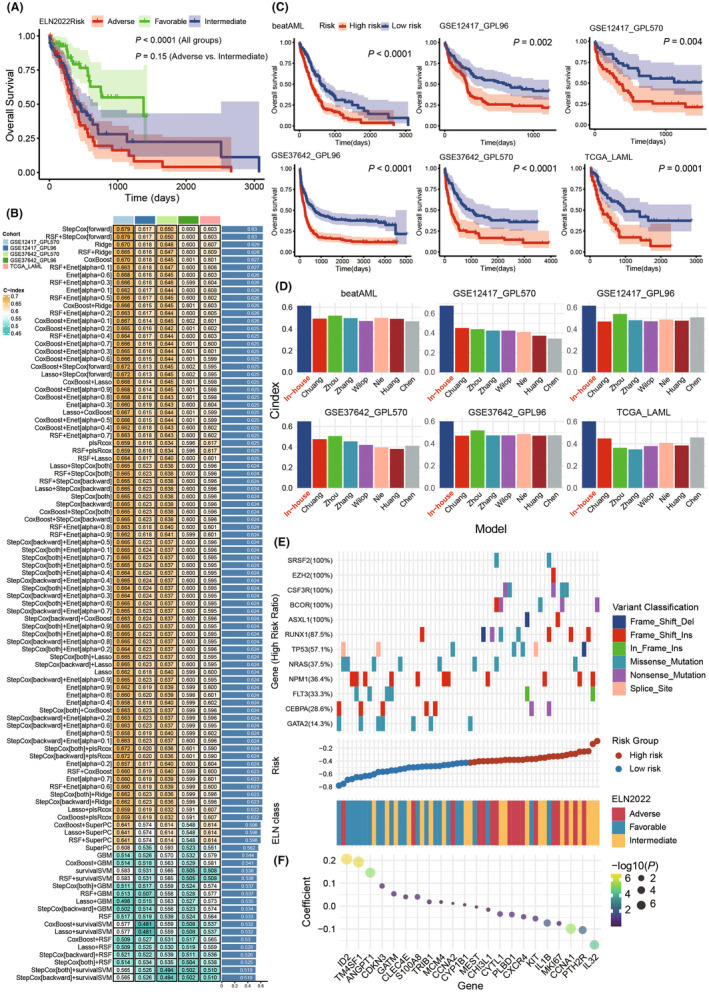
Transcriptomic‐level risk classification in AML and comparative evaluation with Established Models. (A) OS analysis based on ELN2022 stratification in the beatAML dataset. (B) C‐Index for 99 models under the LOOCV framework across validation sets. (C) Risk classification and OS analysis in training and validation sets using the StepCox (forward) model. (D) C‐index with seven published AML models across six datasets. (E) Patient scoring and ELN2022 and genotype distribution in the beatAML dataset using the StepCox (forward) model. (F) Coefficients and *p*‐values corresponding to the StepCox (forward) model.

In our preliminary study, beatAML patients were classified using the ELN2022 classification and overall survival (OS) analyses were conducted (Figure 3A).^34^ While there was a significant difference in OS among different risk groups (*p* < 0.0001), the difference between the adverse and intermediate groups was not significant (*p* = 0.15). This suggests that intermediate group patients may also face poor prognosis, complicating clinical assessments and treatment planning. To address this, we aim to develop an AML risk classification model at the transcriptomic level using our custom‐built machine learning framework, which targets potential therapeutic interventions for high‐risk patients.

The TCGA‐beatAML dataset was designated as the training set before starting our machine‐learning analysis. Using its prognostic data, we performed univariate COX regression analysis on 191 AS, identifying 23 prognostic‐related genes (Figure S6). The C‐index for each of the 99 machine‐learning models across five validation sets was then evaluated. Both the StepCox (forward) algorithm and RSF + StepCox (forward) yielded the highest mean C‐index of 0.63. Due to their comparable performance, we selected StepCox (forward selection) as the primary model, which pinpointed 22 genes (Figure 3B,F). After scoring patients in the training set and validation sets, we divided them into the high‐ and low‐risk groups based on median scores. Survival analysis showed a significantly lower OS for high‐risk patients (Figure 3C). Our model clearly distinguished the 88 samples classified as intermediate risk by ELN2022 stratification into 46 high‐ and 42 low‐risk groups. Survival analysis revealed that the high‐risk group had significantly poorer prognosis compared to the low‐risk group (*p* < 0.05, Figure S7), indicating substantial differences within the intermediate‐risk samples under ELN2022, which could impact clinical decision‐making. The C‐index of our model was compared against seven established AML models, consistently exhibiting better predictive power (Figure 3D; Table S8).

To understand the clinical ramifications of our risk scores, a joint analysis integrating mutation data, risk scores, and ELN2022 categorizations within the beatAML cohort was conducted (Figure 3E). Common mutated genes like *SRSF2*, *EZH2* and *CSF3R* were prevalent in high‐risk patients. In contrast, *NRAS* and *NPM1* appeared more in the low‐risk group. These observations align with the ELN2022 clinical classifications and existing AML clinical research.^35^, ^36^, ^37^, ^38^, ^39^, ^40^, ^41^, ^42^, ^43^, ^44^

### 3.7 Solasonine as a potential therapeutic for high‐risk patients

Utilizing molecular docking simulations, we evaluated the interaction between seven target proteins (*p* < 0.05) from the StepCox (forward) model and 4454 drugs. Drugs with a binding affinity of −5.0 kcal/mol or lower were deemed favourable. Priority was given to drugs consistently ranking in the top 30 for binding affinity. Notably, solasonine demonstrated favourable binding across all seven target proteins (Figure S8 and Table S9). Based on these findings, solasonine emerges as a promising therapeutic candidate for AML.

To further explore solasonine's effects on AML, we analysed RNA‐seq data from the SRP290643^45^ dataset housed in the Sequence Read Archive (SRA, https://www.ncbi.nlm.nih.gov/sra/). This dataset includes both solasonine‐treated and untreated THP‐1 cells. Data processing with DESeq2, using a logFC threshold of 1 and an adjusted *p*‐value of <0.05, identified 2447 DEGs. Comparing these DEGs with a set of 191 AS genes, we discovered 69 common genes. Subsequent GO functional analyses revealed a broad spectrum of solasonine's effects on AML, influencing myeloid cell functions, immune pathways, cellular membrane components and receptor activities. The KEGG enrichment specifically highlighted six major areas of influence (Figures S8B and S9A; Table S10): cellular signalling, genetic regulation, haematopoiesis, immune modulation, innate immune response and microenvironment interaction. Collectively, this evidence showcases solasonine's promising therapeutic potential for AML, and reveals its utility in future clinical interventions.

### 3.8 XGBoost model for identifying cell origins in AML patients versus normal individuals

**FIGURE 4:**
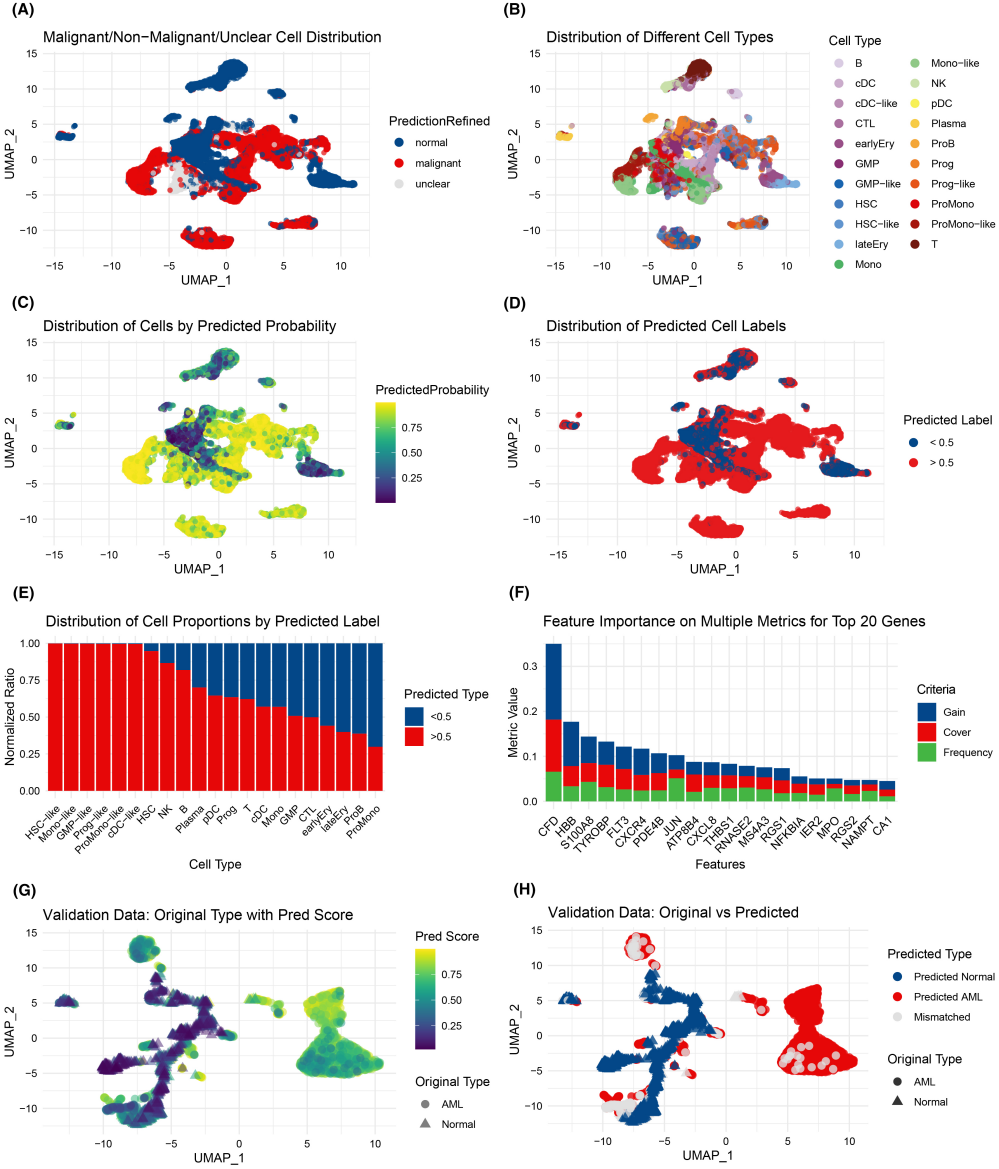
Annotation, classification, and validation of cell origins using single‐cell RNA‐seq data and the XGBoost model. (A) Malignancy level annotation of cells visualized on a UMAP plot sourced from the ABC portal. (B) Cell type annotations visualized on a UMAP plot. (C) Predicted probabilities of cell origin based on the XGBoost model; higher probabilities indicate a greater likelihood of originating from an AML patient. (D) Dichotomous categorization of predicted probabilities with a threshold of 0.5. Cells with probabilities exceeding 0.5 are classified as originating from AML patients. (E) Distribution of cell proportions in different predicted clusters; clusters ending in ‘‐like’” denote malignant cells believed to originate from AML patients. (F) Top 20 significant genes identified by the XGBoost model, ranked based on indicators such as gain, cover, and frequency. (G) Predicted probabilities of cell origins in the GSE198052 dataset. (H) Comparison between predicted and actual cell origins.

To validate the robustness of AML signatures at the single‐cell level, we aim to distinguish cells derived from AML patients from those of healthy individuals using the XGBoost algorithm. We conducted quality control and dimensionality reduction on the GSE116256 single‐cell RNA‐seq data, visualizing the results with annotation information from the ABC portal (Figure 4A and Figure S7B). The XGBoost model predicted cell origins (Figure 4C), classifying cells with a predicted probability above 0.5 as derived from AML patient (Figure 4D). The model effectively identified nearly all malignant cell types, including HSC‐like and mono‐like cells (Figure 4E), and revealed significant variances in cell proportions across different cell types within AML's tumour microenvironment, such as HSC, NK and B cells. These observations align with previous research.^46^

To evaluate the XGBoost results, we used the grain, cover and frequency criteria to identify the top 20 impactful genes, including *CFD*, *HBB* and *S100A8* (Figure 4F).^47^, ^48^, ^49^ We then validated our model with the GSE198052 dataset (Figure 4G,H). The model achieved an AUC of 0.97 for the training set and 0.94 for the validation set, demonstrating high accuracy in distinguishing cells from AML patients and healthy individuals (Figure S9).

### 3.9 Construction of an interactive comprehensive AML signatures database

We developed CSAMLdb, a specialized database for AML research. The database integrates seven core functionalities: gene expression analysis, differential gene scoring, machine learning‐based single‐cell analytics, user‐data integration, pathway enrichment, pharmacogenomic evaluations and literature mining through named entity recognition. Accessible at http://www.csamldb.com, CSAMLdb aims to be a valuable resource for both researchers and clinicians in the field of AML (details in Supplementary Methods).

## 4 DISCUSSION

AML is a highly heterogeneous and aggressive haematological malignancy. Studies have shown that RNA‐Seq is more effective than whole genome and exome sequencing for diagnostic purposes, therefore, it enables precise risk stratification and the selection of targeted therapies for AML patients.^50^, ^51^ Despite several AML signature studies based on individual cohorts and validated against other datasets, their robustness and comprehensive evaluation remain questionable.^35^, ^36^, ^37^, ^38^, ^39^, ^40^, ^41^, ^42^, ^43^, ^44^ Our integrative analysis of 34 AML‐associated datasets revealed substantial transcriptional variability in AML. Nonetheless, we identified and validated 191 consistently expressed AS across multiple datasets as shown in Figure S1. These insights formed the foundation for further prognostic assessment, mechanistic analysis and drug screening for AML, illustrated in Figure 1.

Regarding prognostic analysis, the updated European LeukemiaNet's ELN2022 risk stratification system for adult AML show slightly lower prognostic efficacy for overall survival and similar predictive performance for complete remission, relapse rates and disease‐free survival compared to its ELN2017 predecessor.^34^, ^52^, ^53^ Our observations align with the trends noted in ELN2022, supporting our preliminary findings. Our model, constructed from an array of 99 machine learning algorithms, has demonstrated superior predictive performance across five independent validation datasets, surpassing existing research models in prognostic accuracy. Integrating our risk scores with the ELN2022 stratification and mutational profiles, this model adheres to ELN2022 standards while providing a more detailed subdivision of the intermediate risk category into the high‐ and low‐risk subgroups. Mutation analysis revealed a predominance of deleterious mutations in high‐risk patients (above 50%), while protective mutations were more common in the low‐risk patients. In particular, among patients with *FLT3* mutations, those with in‐frame insertions had worse outcomes, whereas those with missense mutations showed a reduced risk, validating previous studies on *FLT3* mutation impacts.^54^ This indicates that out model not only refines risk stratification but also enhances its clinical predictive value across multiple dimensions.

Our objective extends beyond prognostic predictionto align more closely with clinical requirements by forecasting potential therapeutic drugs for high‐risk patients. Molecular docking was performed between seven prognosis‐related genes (*CCAN1*, *PTH2R*, *ID2*, *IL32*, *ANGPT1*, *IL1B*, *TM4SF1*) identified by our machine learning model and small molecule drugs. Notably, solasonine exhibited strong binding affinity with all seven molecules. Extracted from the herbal plant *Solanum nigrum* Linn., solasonine is a steroidal alkaloid with potential anti‐tumour effects against various cancers, including pancreatic, gastric and lung adenocarcinoma. Although solasonine's therapeutic application in AML remains underexplored, these findings suggest its potential use. If further validated, patients might benefit from a new treatment option targeting previously unexplored mechanisms.^55^, ^56^

In this study, an examination of DEGs from the intersection of solasonine and AS highlighted significant enrichment in pathways such as cellular signalling and growth control, genetic and transcriptional regulation, and others related to haematopoiesis, immune response, and microenvironment interaction. This emphasizes the intricate interplay of these molecular mechanisms and hints at solasonine's potential in AML. Therefore, in‐depth exploration of these mechanisms is imperative to ascertain solasonine's exact roles and therapeutic potential.

ITo enhance the precision of AML cell origin identification, we employed an XGBoost model, which significantly advanced our approach. While platforms like ScType have contributed to personalized AML treatment strategies,^57^ they require an extensive marker database, potentially limiting rapid analysis. In contrast, our model relies solely on the scRNA‐seq dataset, ensuring swift and precise cell origin identification. This approach expedites the formulation of personalized therapeutic regimens, which can lead to tailored treatments for AML patients, potentially reducing side effects and improving response rates.

Moreover, integrating our machine learning model with inferCNV's capacity to detect malignancy via copy number variation streamlines cell categorization and lays a solid foundation for discerning malignancy within AML (Figure 4A–D). This integration expands the prospects for more efficient and time‐saving personalized therapeutic strategies.

Previous studies have established several valuable AML‐related databases:

1. www.leylab.org/amlproteome, which primarily compiles proteomic and mRNA data from 44 AML samples in TCGA but has a small sample size that may limit generalizability.^58^
2. BloodChIP Xtra (http://bloodchipxtra.vafaeelab.com/), which includes data on chromatin accessibility and gene expression in primary human haematopoietic stem/progenitor cells and AML cells, but lacks patient‐level validation.^59^
3. BloodSpot (https://bloodspot.eu/), which provides gene expression data in healthy and malignant haematopoiesis.^60^ However, like the first two databases, it mainly displays and aggregates single data types (individual genes/proteins), which may be insufficient for researchers needing personalized analysis based on clinical and experimental data.

Building on our previous work, we have introduced CSAMLdb, a novel and comprehensive database platform dedicated to AML signatures. Designed for both researchers and clinicians, this platform boasts seven core features: online prognostic analysis, multi‐gene scoring, single‐cell score and classification, data matching, enrichment analysis, drug prediction, risk scoring and literature entity recognition (Figure 1, accessible at http://www.csamldb.com/). These features not only offer potential clinical applications but also present opportunities for further scientific exploration and reuse in subsequent studies.

In conclusion, our study has led to the creation of CSAMLdb, a comprehensive AML database that provides robust AML signatures, a high‐performance prognostic model, and multi‐pathway drug effects analysis, including solasonine's impact on AML. This user‐friendly platform is designed to advance clinical practice and foster scientific discovery, offering new avenues for the study and treatment of AML.

## PERMISSION TO REPRODUCE MATERIAL

All materials presented in this manuscript are either original or have been sourced from public databases with appropriate usage rights.

## AUTHOR CONTRIBUTIONS

**Haoran Chen:** Conceptualization (lead); data curation (lead); formal analysis (lead); project administration (lead); resources (lead); writing – original draft (lead); writing – review and editing (lead). **Jinqi Lu:** Conceptualization (equal); data curation (lead); software (equal); writing – review and editing (equal). **Zining Wang:** Conceptualization (equal); supervision (lead); validation (lead); writing – review and editing (lead). **Shengnan Wu:** Formal analysis (equal); investigation (equal); methodology (equal); validation (equal). **Shengxiao Zhang:** Supervision (equal); writing – review and editing (lead). **Jie Geng:** Methodology (equal); resources (equal); software (equal); visualization (equal). **Chuandong Hou:** Conceptualization (supporting); formal analysis (equal); validation (equal). **Peifeng He:** Conceptualization (lead); funding acquisition (lead); project administration (lead); supervision (lead); writing – review and editing (lead). **Xuechun Lu:** Conceptualization (lead); funding acquisition (lead); project administration (lead); supervision (lead); writing – review and editing (lead).

## FUNDING INFORMATION

This work was supported by the National Social Science Fund of China [21BTQ050]; the Key R&D Program of Shanxi Province “Research on Key Technologies of Multi‐source Data Drug Repositioning” [202102130501003]; National Key R&D Program of China [2020YFC2002706]; Sub‐project of National Key R&D Program of China [2021YFC2701704‐1]; Multi‐center Clinical Research Project of National Clinical Research Center for Geriatric Diseases (Chinese PLA General Hospital) [NCRCG‐PLAGH‐20230010]; Key Military Health Project [23BJZ25]; Clinical Decision‐Making Research Big Data Shanxi Province Key Laboratory [2021D100012021515245001135236]; National Natural Science Foundation of China [82001740]; and the Natural Science Foundation of Shanxi Province [202203021221269].

## CONFLICT OF INTEREST STATEMENT

None declared.

## Supporting information


Data S1:

Data S2:

## Data Availability

Data and Code AvailabilityCSAMLdb is accessible to the public at http://www.csamldb.com. All metadata in the database can be publicly accessed at https://download.csamldb.com. Our research code can be found on GitHub: https://github.com/CHR991004/AML_Pipeline.
