# Supplementary material for: Unlocking reproducible transcriptomic signatures for acute myeloid leukaemia: Integration, classification and drug repurposing
Source: J Cell Mol Med. 2024 Sep 12;28(17):e70085. doi: 10.1111/jcmm.70085 (PMC11392829; doi:10.1111/jcmm.70085)
Supplement: Supplementary file 1 — Data S1: [file JCMM-28-e70085-s001.docx]

**Unlocking Reproducible Transcriptomic Signatures for Acute Myeloid Leukemia: Integration, Classification, and Drug Repurposing**

Haoran Chen^1,2,3†^, Jinqi Lu^4†^, Zining Wang^5,6†^, Shengnan Wu^2^, Shengxiao Zhang^7,8^, Jie Geng^9^, Chuandong Hou^5,6^, Peifeng He^2,10*^ and Xuechun Lu^2,3,5*^

^1^ School of Biomedical Engineering and Informatics, Nanjing Medical University, Nanjing, China.

^2^ School of Management, Shanxi Medical University, Taiyuan, 030000, China.

^3^ Department of Nephrology, First Medical Center of Chinese PLA General Hospital, Nephrology Institute of the Chinese People’s Liberation Army, National Key Laboratory of Kidney Diseases, National Clinical Research Center for Kidney Diseases, Beijing Key Laboratory of Kidney Disease Research, Beijing 100853, China

^4^ Department of Computer Science, Boston University 665 Commonwealth Avenue, Boston, Massachusetts, 02215, USA

^5^ Department of Hematology, The Second Medical Center of Chinese PLA General Hospital, National Clinical Research Center for Geriatric Disease, Beijing, 100853, China

^6^ Medical School of Chinese PLA, Beijing, 100853, China

^7^ Department of Rheumatology and Immunology, the Second Hospital of Shanxi Medical University, Taiyuan, China
^8^ Key Laboratory of Coal Environmental Pathogenicity and Prevention at Shanxi Medical University, Ministry of Education, Shanxi Province, Taiyuan, China
^9^ Basic Medicine College, Shanxi Medical University, Taiyuan, 030000, China

^10^ Shanxi Key Laboratory of Big Data for Clinical Decision, Shanxi Medical University, Taiyuan, 030000, China.

* To whom correspondence should be addressed.

† The authors wish it to be known that, in their opinion, the first three authors should be regarded as Joint First Authors.

**Email:** **Xuechun Lu**: luxuechun@301hospital.com.cn; **Peifeng He:** [hepeifeng2006@126.com](mailto:hepeifeng2006@126.com)

# Supplementary Methods

## Data Sources

To prepare files for differential gene expression analysis, we used the following retrieval strategy: “leukemia, myeloid, acute” [MeSH Terms] OR acute myelogenous leukemia; [All Fields] AND “bone marrow” [MeSH Terms]; OR bone marrow [All Fields] AND (“gse” [Filter] AND (“Expression profiling by array” [Filter]; OR “Expression profiling by high throughput sequencing” [Filter]. The search was conducted in the GEO database and further filtering was used to select datasets with at least three replicates in both the AML group and the normal control group.

To prepare files for prognostic analysis, we included AML studies with prognostic information in both the GEO and TCGA databases. To prepare files for single-cell RNA sequencing (scRNA-seq) analysis, we retrieved single cell sequencing data for newly diagnosed AML patients from GEO. It was necessary to include normal cells and have clear barcode annotations.

## Standard Transcriptome Data Analysis

Differential expression analysis was carried out using different, but standardized processes, for various AML datasets. The threshold for differentially expressed genes across all datasets was set to *P* < 0.05，|log2FC| > |log2FC|_mean_ + 2 × |log2FC|_SD_, where log2FC represents the log2-fold change in gene expression. In addition, RNA-seq expression data obtained from high throughput sequencing were quantified using the standardized workflow from the GREIN database^1^(http://www.ilincs.org/apps/grein). Differential expression analysis was performed using the DESeq2(version 1.40.2) package in R. For microarray data^2^, we obtained the data from the GEO database and processed it using the limma(version 3.56.2) package^3^.

To ensure the model's applicability across diverse datasets, we transformed the raw count matrix into FPKM values, followed by imputation of missing values, quantile normalization, and log2 transformation.

## Filtering and Evaluation of AML Comprehensive Signatures

To identify AML-related genes (ARGs), we selected genes that appeared at least once in all of the differential analysis results from the RNA-seq datasets. Because there were too many possible combinations for all 26 studies, we repeated the sampling process four times. For each sampling, we selected 12 studies and calculated the average Gene Mean Consistency Score (*GMCS*) and coefficient of variation (*CV*) of gene expression for each combination of the 12 studies. This was done to determine the consistency and variability of ARGs and generate the AS. The formula was as follows:

$$GMCS= \frac{\sum_{i=1}^{n} {count}_{i}}{n}$$

$$CV= \frac{{mean}_{EXP}}{{SD}_{EXP}}$$

*n*: The number of genes in the combined dataset(s).

*count*: The frequency with which a gene in the combination appears across the 26 datasets.

*EXP:* Gene expression levels within the combined dataset(s).

## Evaluation of AML Dataset Similarity

To determine the similarity between different datasets and the AS, we used radial graphs. This was necessary because of the varying number of differentially expressed genes and the differences in similarity with AS in various datasets. Similarity is evaluated based on the proportion of overlapping genes between an AML dataset and AS. The formula was as follows:

$$similarity= \frac{IG}{DG}$$

*IG*: Intersecting Genes. The intersection genes between AS and an AML dataset.

*DG:* Disease Genes. All genes in single AML dataset.

## Gene Set Rank Score

Considering the different number of occurrences of different genes in the study, the number of occurrences was used as a weight and the score of different genes in different AML datasets was evaluated with a rank score. The formula was as follows:

$$RS=\sum_{i=1}^{n(ARG)} \left\{ \begin{aligned} &S_{i} &&S_{i}>1 \\ &-1 &&S_{i}=1 \end{aligned} \right.$$

*S_i_:* Frequency of differential genes appearing in 26 datasets

*ARG*: AML related genes.

*n(ARG)* = 10000

## Disease Gene Signature Enrichment Score

In the proposed method, the Disease Gene Signature Enrichment (DGSE) score is computed for each individual gene position in the ranked list of differentially expressed genes (DEG). The cumulative DGSE score for the dataset is then calculated by iteratively applying this formula for each gene position in the ranked list and selecting the maximum value obtained. Specifically, the DGSE score is given by the formula:

$$DGSE= \frac{n\left( AS\cap DEG \right)-\frac{n(DEG)\times n(AS)}{n(ARG)}}{n(DEG)}$$

$n\left( AS\cap DEG \right)$: The count of genes that are both in the AS and the ranked list of DEG

$\frac{n(DEG)\times n(AS)}{n(ARG)}$: Serves as a normalization factor to adjust for the background noise introduced by the size of the AML-related genes (ARG).

$n(DEG)$: The number of differentially expressed genes in the ranked list.

For illustrative purposes, let's consider a scenario in which the dataset of differentially expressed genes (DEG), ranked by importance according to logFC, *P*-value, or adjusted *P*-value, contains the following genes: *CLU, IFITM1, HBEGF, BASP1, CYP1B1, AHR,* and *F9*. Among these, *CLU, IFITM1, HBEGF, BASP1,* and *CYP1B1* are part of the AS. *AHR* appears 5 times, while *F9* is also not part of AL and appears 0 times. Additionally, the AS comprises a total of 191 genes (times ≥ 6), and the AL includes 10,000 genes (times ≥ 1). The DGSE score for individual genes can be calculated as follows:

Highest score observed at *CYP1B1*:

$$DGSE=\frac{(5-\frac{7\times191}{10000})}{7}=0.9809$$

Subsequent reduction in score at *AHR*:

$$DGSE=\frac{(5-\frac{6\times191}{10000})}{6}\approx0.8142$$

The calculation result for RS is 12(*CLU*) + 11(*IFITM1*) + 11(*HBEGF*) + 10(BASP1) + 10(*CYP1B1*) + 5(*AHR*) – 1 = 58

In summary, the DGSE primarily serves as a qualitative measure to evaluate the proportion of key genes (AS) that appear in a dataset's DEG. On the other hand, the Rank Score (RS) provides a quantitative assessment of the cumulative frequency of DEGs.

## DGIdb-based Drug Enrichment and Repurposing

The Drug-Gene Interaction Database (DGIdb) is a rigorously curated resource that aggregates known drug-gene interactions from multiple authoritative sources^4^. This extensive compilation offers crucial insights into well-established drug-target associations. Leveraging the associations within DGIdb, we identified potential therapeutic agents correlated with the AML signatures. Subsequently, a drug enrichment analysis was conducted, honing in on ATC (Anatomical Therapeutic Chemical) level 3 codes, which spotlight specific therapeutic and pharmacological subgroups. This analysis provides a clearer understanding of the functional roles of these associated drugs. The enrichment process is facilitated by our dedicated DrugTargetEnrich database, accessible at <https://bioinfordatascience.shinyapps.io/DrugTargetEnrich/>.

## Construction of the Protein-Protein Interaction (PPI) Network.

To elucidate the interconnectivity and key nodes within the AML signatures, we constructed interaction networks using the STRING (version 12.0) database^5^ and visualized them with Cytoscape (version 3.9.0) software^6^. We set our parameters to focus on Homo sapiens, with an interaction score cutoff of 0.4. After obtaining the interaction data, we employed the CytoHubba plugin in Cytoscape to refine the network's structure^7^. The MCC (Maximal Clique Centrality) algorithm, known for accurately identifying densely connected nodes in complex networks, helped us pinpoint a key set of the top 10 core genes, streamlining our subsequent analyses. Additionally, we employed the survival package (version 3.5-5) to assess the survival implications of these 10 hub genes in relation to prognostic outcomes.

## Non-negative matrix factorization for clustering

To identify distinct AML subtypes, we applied NMF clustering to 191 AML signatures from 213 AML samples in the TCGA-beatAML dataset using the ConsensusClusterPlus package in R^8^. For enhanced stratification reliability, in each of the 50 iterations, we randomly selected 80% of the original samples as subsamples, ensuring all features were retained. These subsamples were then categorized into up to 10 clusters using the k-means algorithm with the Pearson distance metric. The optimal cluster number (K-value) was ascertained via the Cumulative Distribution Function (CDF) curve, with a more stable curve indicating a more consistent clustering outcome. Using the pheatmap (1.0.12) package^9^, we visualized the expression patterns of the 191 AML signatures across the identified subtypes. Additionally, survival analyses were conducted among these AML subtypes.

## Optimization of Machine Learning Algorithms for AML Prognostic Modeling

In our prognostic signature discovery, we harnessed an array of machine learning algorithms, each chosen for its strengths in analyzing high-dimensional survival data. For instance, the Random Survival Forests (RSF) were operationalized using the randomForestSRC package, prized for its robust handling of censored data through an ensemble of de-correlated decision trees. The RSF's hyperparameters—ntree, the number of trees, and mtry, the subset of variables considered at each split—were optimized through a meticulous grid-search within a Leave-One-Out Cross-Validation (LOOCV) framework, identifying the combination yielding the highest concordance index.

In parallel, Elastic Net, Lasso, and Ridge regression models were implemented via the glmnet package, each bringing a distinct edge to variable selection and multicollinearity adjustment. Elastic Net's dual regularization parameters—the number of variables (λ) and the L1-L2 mixing parameter (α)—were fine-tuned through LOOCV to balance selection accuracy and model complexity.

Stepwise Cox Regression, executed through the survival package, streamlined model complexity by employing an Akaike information criterion (AIC)-driven stepwise algorithm, which intelligently navigated through "both" directions for variable inclusion or exclusion.

CoxBoost, facilitated by the CoxBoost package, focused on incrementally optimizing the Cox proportional hazards model. Its tuning process, governed by LOOCV, first pinpointed the optimal penalty parameter before the cv.CoxBoost function iteratively selected the number of boosting steps, culminating in a dimensionality-defined model through the principal CoxBoost routine.

The plsRcox package brought Partial Least Squares Regression to our suite, applying the cv.plsRcox function to ascertain the optimal number of components, followed by the plsRcox function to fit the model.

Supervised Principal Component Analysis (SuperPC), operational through the superpc package, crafted a predictive model via a supervised version of principal component analysis, determining the optimal feature threshold through a specialized form of LOOCV, known as "pre-validation," to address small dataset challenges.

Lastly, the Generalized Boosted Model (GBM) and Survival Support Vector Machine (survival-SVM) models were implemented via their respective packages, gbm and survivalsvm, with LOOCV guiding the selection of hyperparameters such as the number of trees for GBM and the optimal constraints for survival-SVM.

## Details in CSAMLdb: A Comprehensive Signatures of AML Database

The CSAMLdb database, constructed on the foundation of this study, is designed for the reuse and reapplication of data and results, encompassing eight distinct functionalities (Figure 1):

1. Single Gene: This section presents single gene analysis within AML datasets, depicting differential expression across various datasets and assessing the prognostic impact of genes using survival analysis in select databases upon gene symbol entry.
2. Multi-Genes: Dedicated to multi-gene analysis, this area allows users to input gene sets for scoring against AML gene sets in the database, identifying pivotal genes within the sets.
3. Single Cell Score and Classification: Utilizing the XGBoost machine learning algorithm, this page scores single-cell expression data against a pre-trained AML transcriptome dataset, offering predictions on the cell's origin and classification, with visual reproduction capabilities based on a reference dataset.
4. Data Matching: An optional feature for those with AML datasets, enabling correlation assessment with reference datasets to inform enrichment analyses and drug prediction, recommending the most correlated dataset as a benchmark.
5. Genes Enrich: This computational method identifies dysregulated functional categories or pathways, utilizing 26 AML datasets, four pathway gene sets, and two algorithms for comprehensive gene enrichment analysis.
6. Drug Prediction: Offering information on a vast array of drugs, this page uses five algorithms to predict potential therapeutics within 26 AML datasets, streamlining the experimental scope and cost.
7. Risk Score: Integrates an optimal algorithm from an analysis of 99 machine learning methods, scoring AML patients within a specified expression spectrum matrix.
8. AI Extract: Compiling AML-related literature from 1976 to 2023, this function employs advanced NER models to extract pertinent information, supporting gene function analysis and the validation and reuse of research findings.

## Reference

1. Mahi NA, Najafabadi MF, Pilarczyk M, Kouril M, Medvedovic M. GREIN: An Interactive Web Platform for Re-analyzing GEO RNA-seq Data. *Sci Rep.* 2019;9(1):7580.

2. Love MI, Huber W, Anders SJGb. Moderated estimation of fold change and dispersion for RNA-seq data with DESeq2. 2014;15(12):1-21.

3. Ritchie ME, Phipson B, Wu D, et al. limma powers differential expression analyses for RNA-sequencing and microarray studies. 2015;43(7):e47-e47.

4. Freshour SL, Kiwala S, Cotto KC, et al. Integration of the Drug–Gene Interaction Database (DGIdb 4.0) with open crowdsource efforts. 2021;49(D1):D1144-D1151.

5. Szklarczyk D, Kirsch R, Koutrouli M, et al. The STRING database in 2023: protein–protein association networks and functional enrichment analyses for any sequenced genome of interest. 2023;51(D1):D638-D646.

6. Shannon P, Markiel A, Ozier O, et al. Cytoscape: a software environment for integrated models of biomolecular interaction networks. 2003;13(11):2498-2504.

7. Chin C-H, Chen S-H, Wu H-H, Ho C-W, Ko M-T, Lin C-YJBsb. cytoHubba: identifying hub objects and sub-networks from complex interactome. 2014;8(4):1-7.

8. Wilkerson MD, Hayes DNJB. ConsensusClusterPlus: a class discovery tool with confidence assessments and item tracking. 2010;26(12):1572-1573.

9. Kolde R, Kolde MRJRp. Package ‘pheatmap’. 2015;1(7):790.

# Supplementary Figures


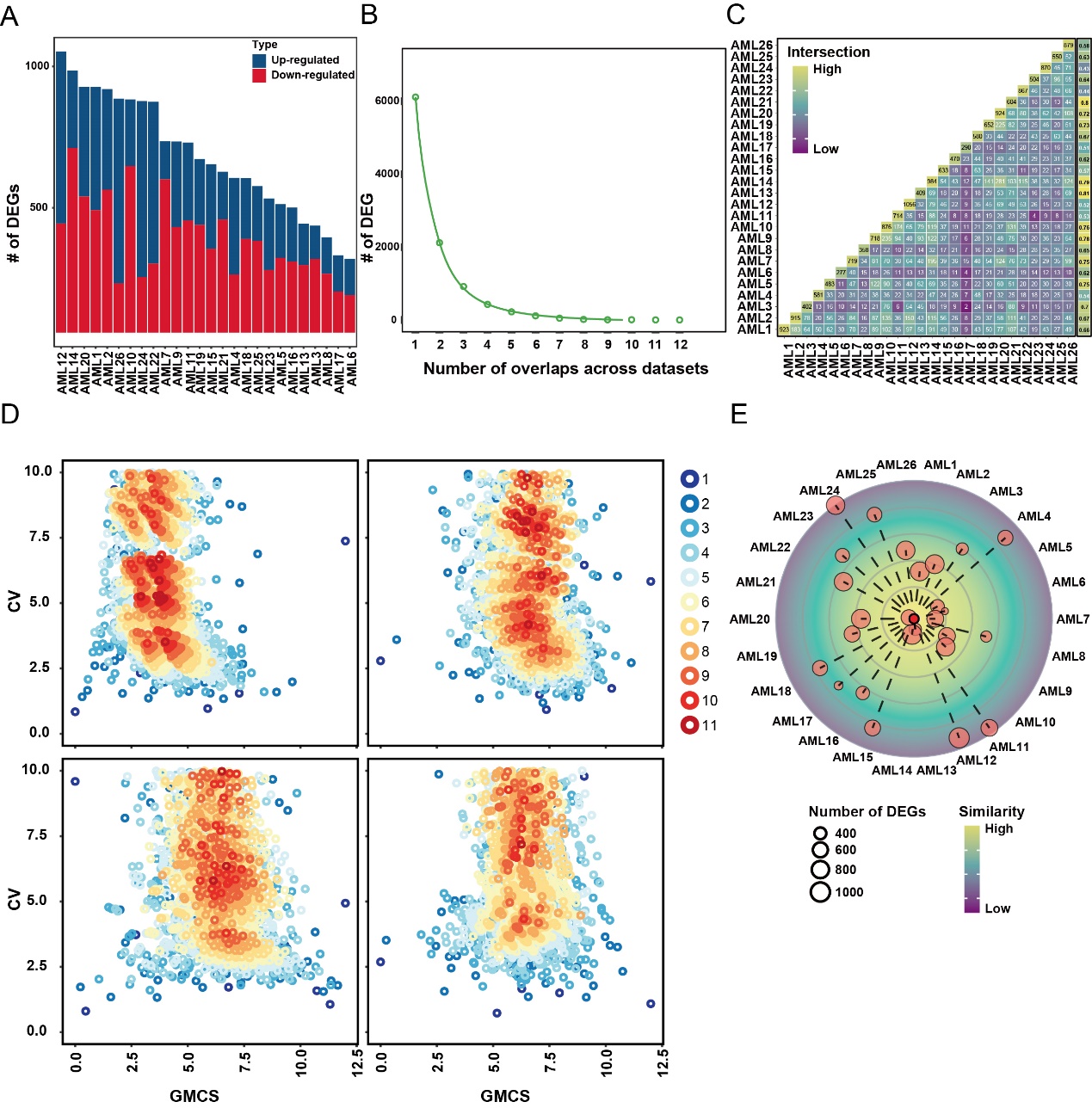


**Supplementary Figure 1. AML consistency gene filtering and evaluation. (A)** Distribution of DEG quantity among 26 datasets. **(B)** Occurrence of DEGs in multiple datasets. The green dot represents the number of differential genes (y-axis) and the number of times these genes appear in the dataset (x-axis) **(C)** Heatmap of shared DEGs between pairwise datasets. The more yellow the color, the greater the number of shared genes; conversely, the fewer shared genes. The right column indicates the proportion of DEGs in the dataset compared with all other datasets. **(D)** By evaluating the consistency of the GMCS and CV datasets with the inclusion of more datasets, the GMCS tends to become consistent and the CV gradually becomes more concentrated. **(E)** Assess the similarity between DEGs and AML signatures in each dataset based on 191 AML signatures.


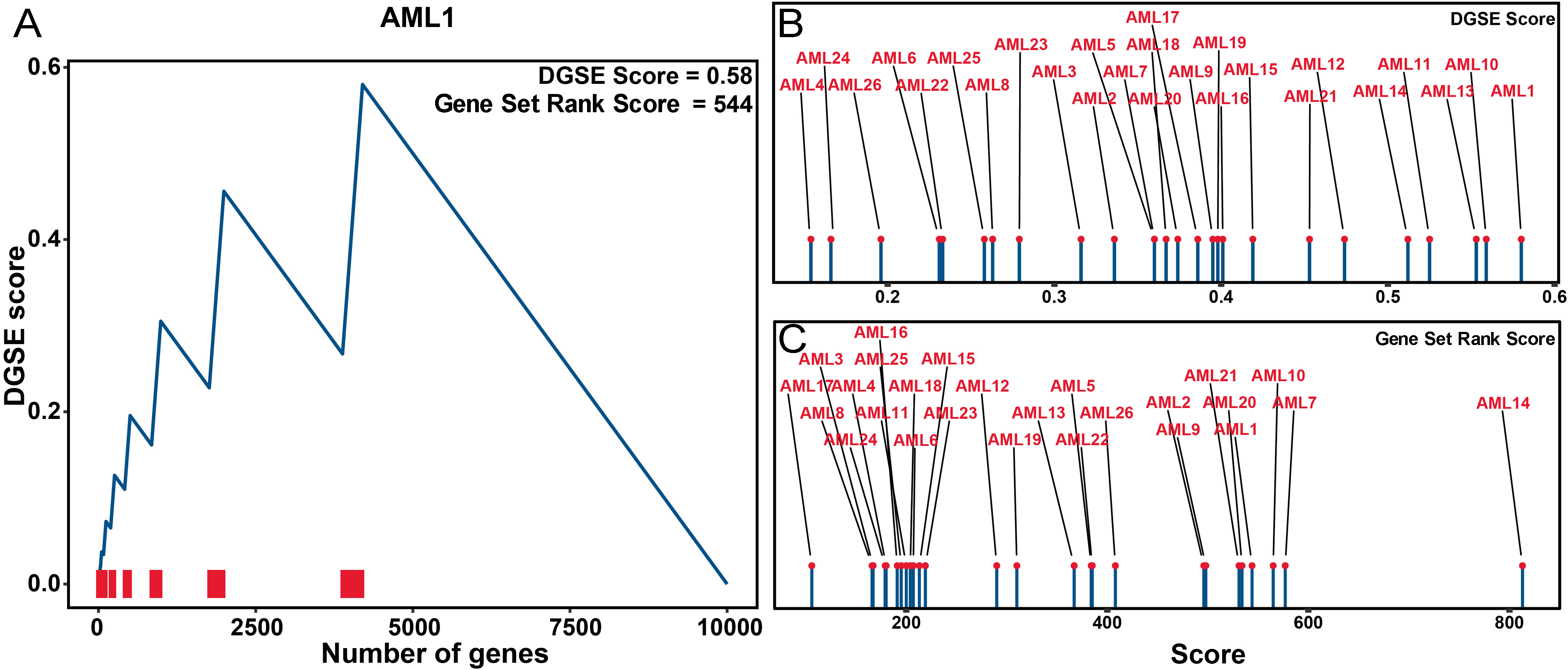


**Supplementary Figure 2. Comparative Analysis of DGSE and RS Algorithms Across Multiple AML Datasets. (A)** DGSE analysis on AML1, highest score as final. **(B-C)** Scores from DGSE and RS algorithm analyses across 26 AML datasets.


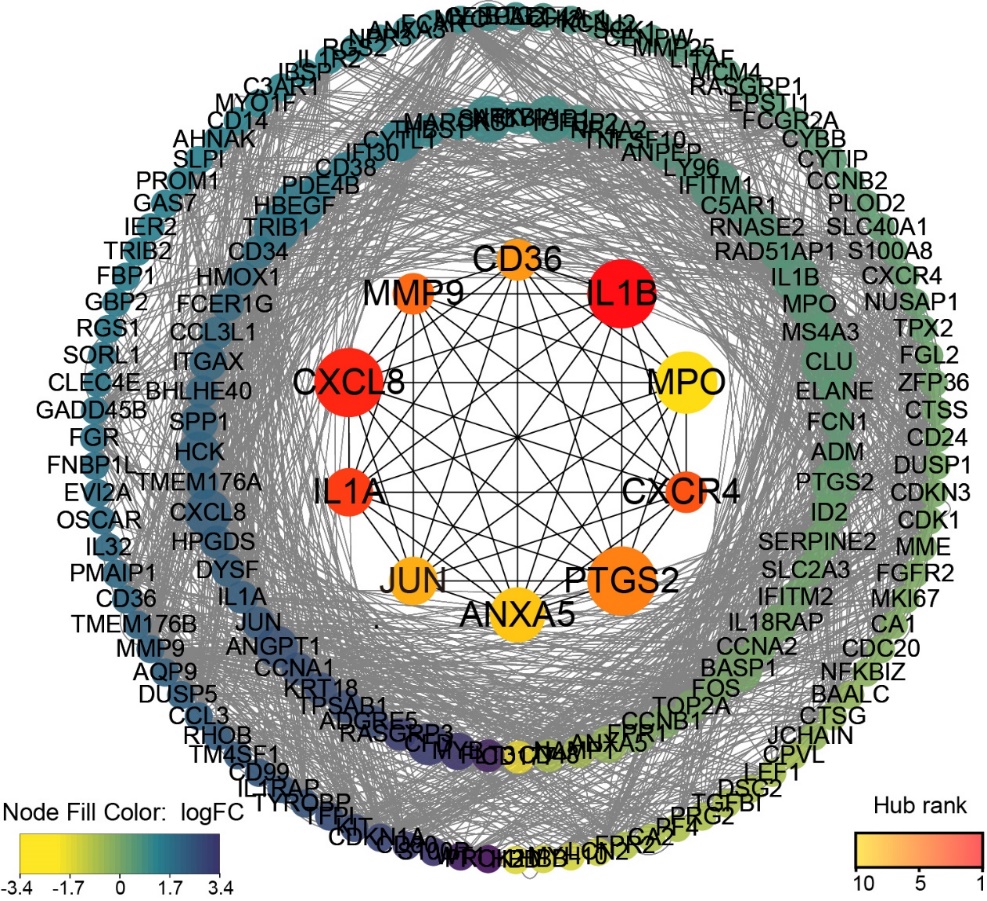


**Supplementary Figure 3.** PPI network of 191 AML signatures with 1217 edges. Inner circle shows 10 hub genes identified by MCC algorithm.


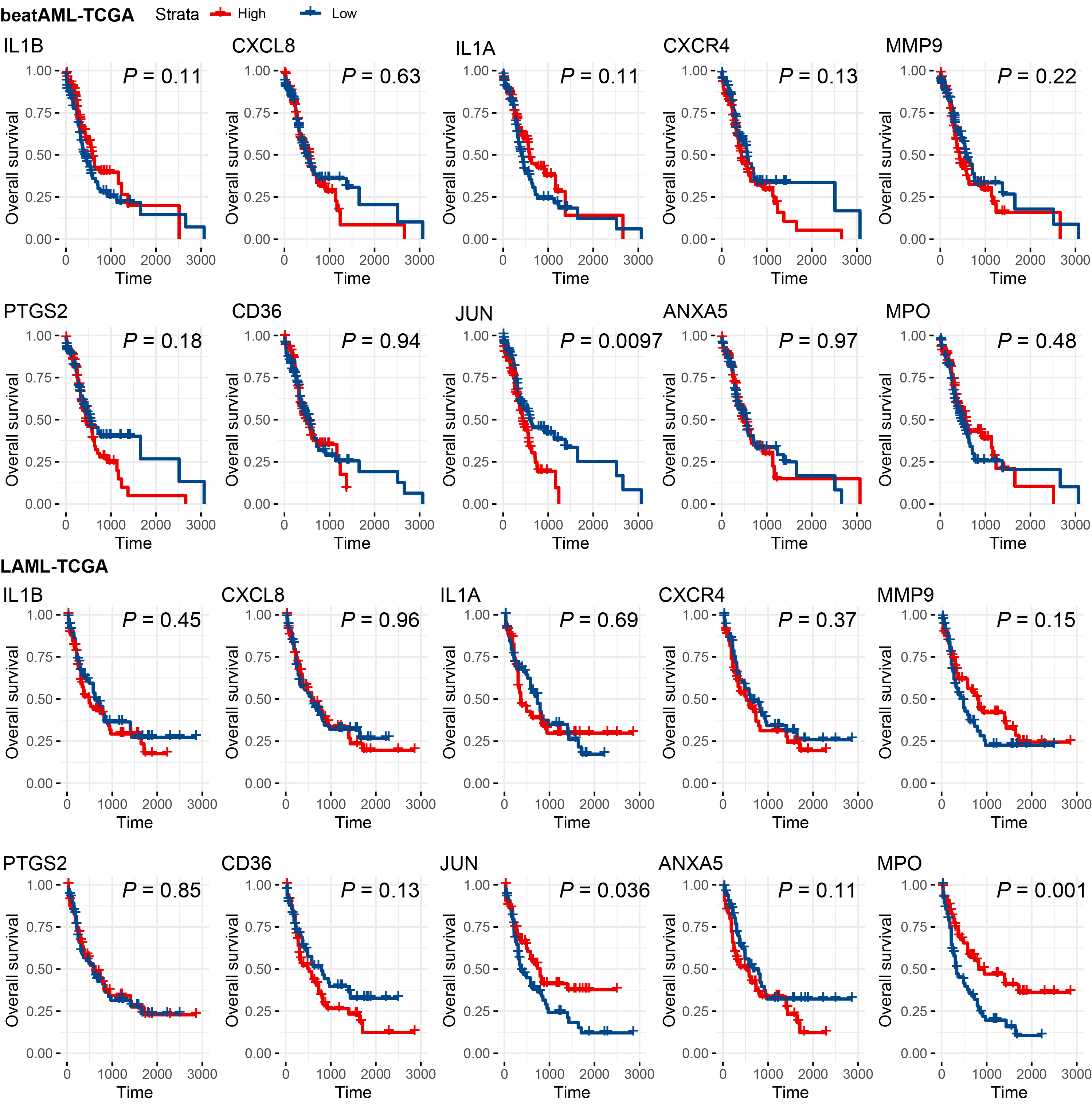


**Supplementary Figure 4.** Overall Survival curves for high and low expression of 10 hub genes in TCGA-beatAML and TCGA-LAML cohorts.


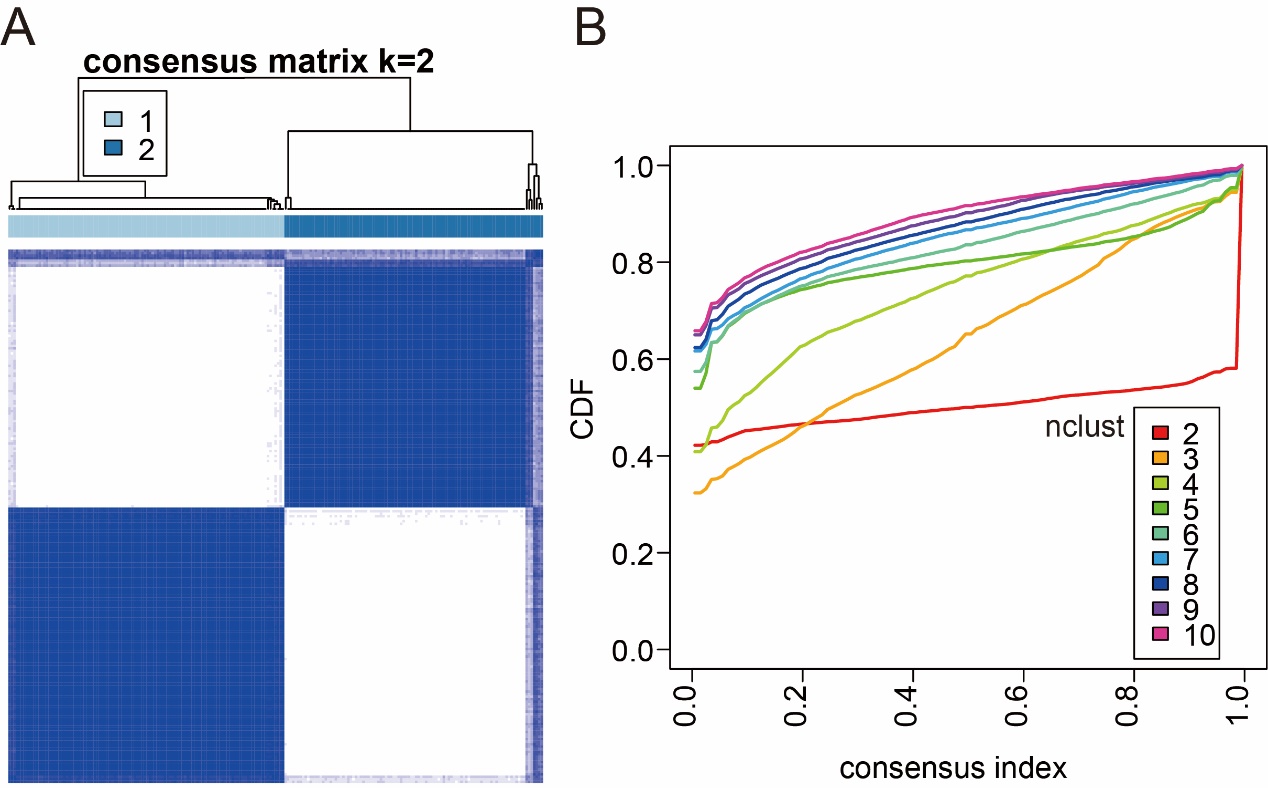


**Supplementary Figure 5. (A)** Consensus matrix resulting from NMF clustering for k=2. The matrix showcases the stratification of 213 AML samples from the TCGA-beatAML dataset into two distinct subtypes based on 191 AML signatures. Each cell in the matrix represents the consensus between samples over 50 iterations, where dark cells indicate a higher consensus that samples belong to the same cluster, and lighter cells indicate a lower consensus. **(B)** Cumulative Distribution Function (CDF) plot for consensus clustering results. The x-axis represents the consensus index, and the y-axis shows the corresponding CDF values. Each curve corresponds to a different number of clusters (k-values) with the curve for k=2 being the lowest and most stable, highlighting its optimal clustering efficacy.


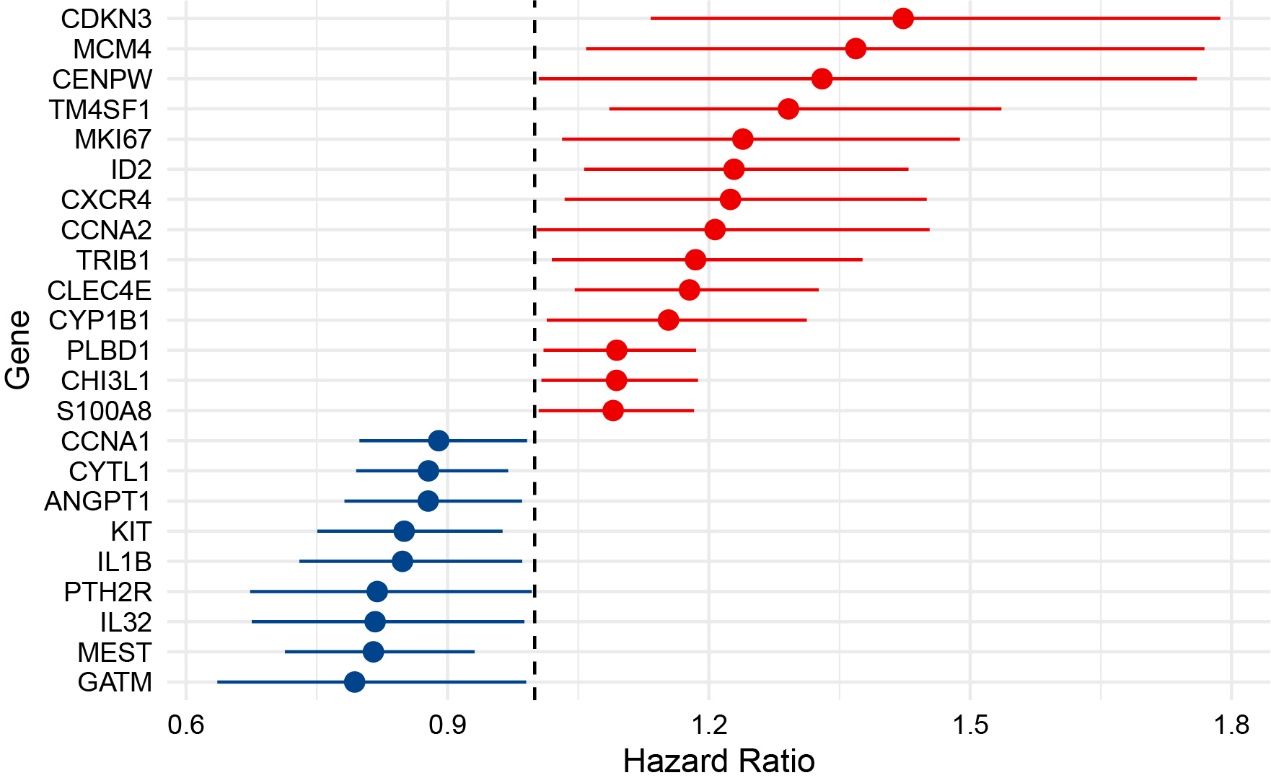


**Supplementary Figure 6.** Forest plot of the univariate Cox regression analysis. The x-axis represents the Hazard Ratio (HR), while the y-axis enumerates the genes analysed. An HR greater than 1 signifies a risk factor, whereas an HR less than 1 indicates a protective factor.


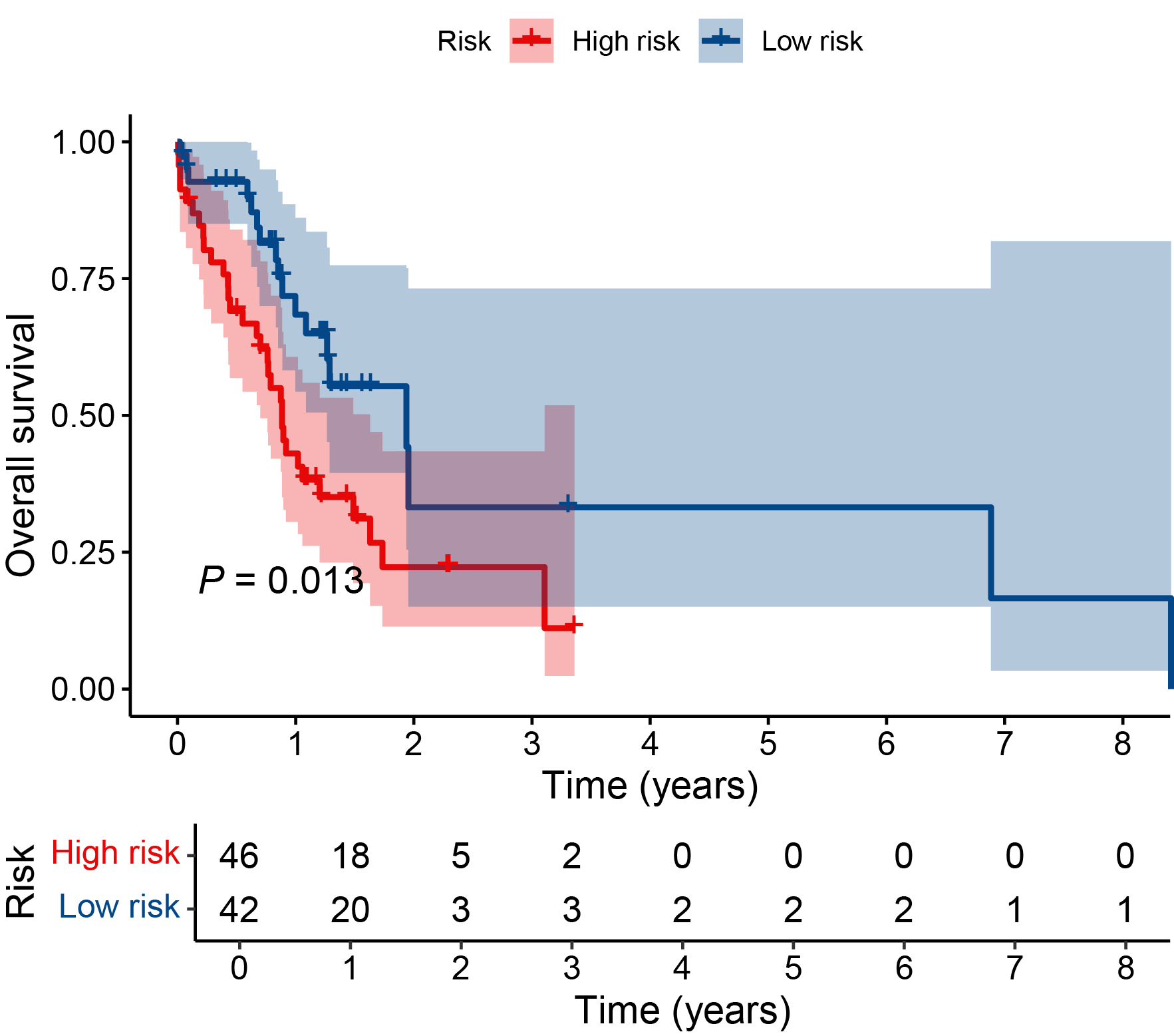


**Supplementary Figure 7.** Survival Curves for High-Risk and Low-Risk Groups in Intermediate ELN2022 Stratification


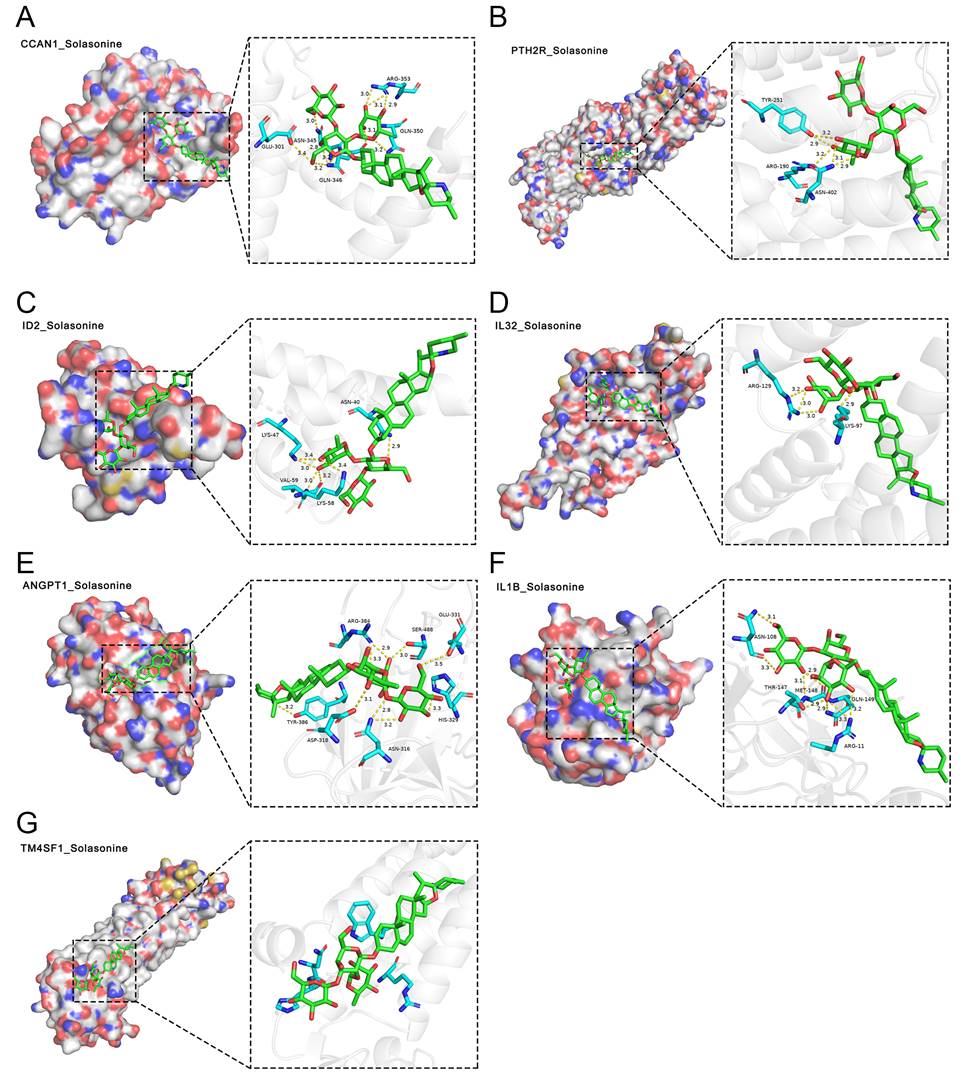


**Supplementary Figure 8.** Binding sites and hydrogen bonds of solasonine with **(A)** CCNA1, **(B)** PTH2R, **(C)** ID2, **(D)** IL32, **(E)** ANGPT1, **(F)** IL1B, and **(G)** TM4SF1.


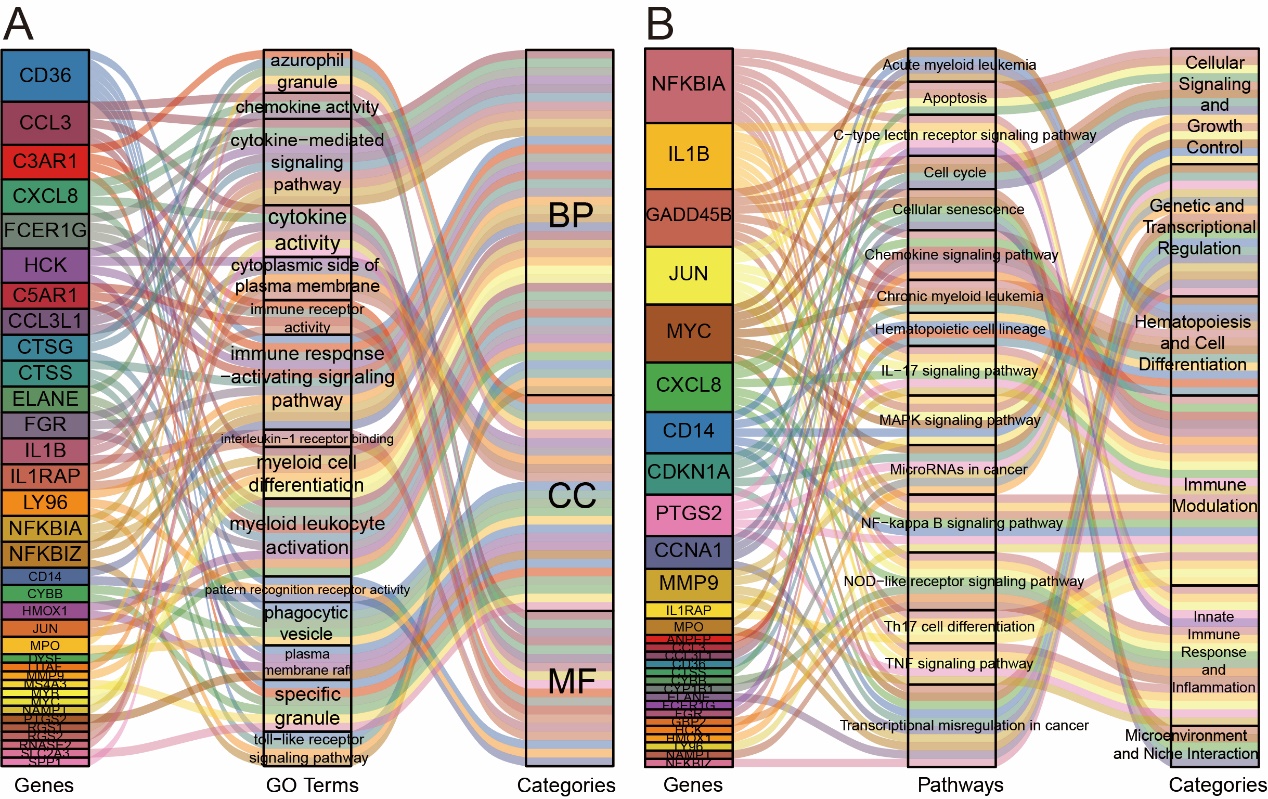


**Supplementary Figure 8. Sankey Diagram of GO and KEGG Enrichment Analyses. (A)** Mapping of Genes to Corresponding GO Terms and Functional Categories. **(B)** Mapping of Genes to KEGG Pathways, with Categories Manually Annotated by Experts.


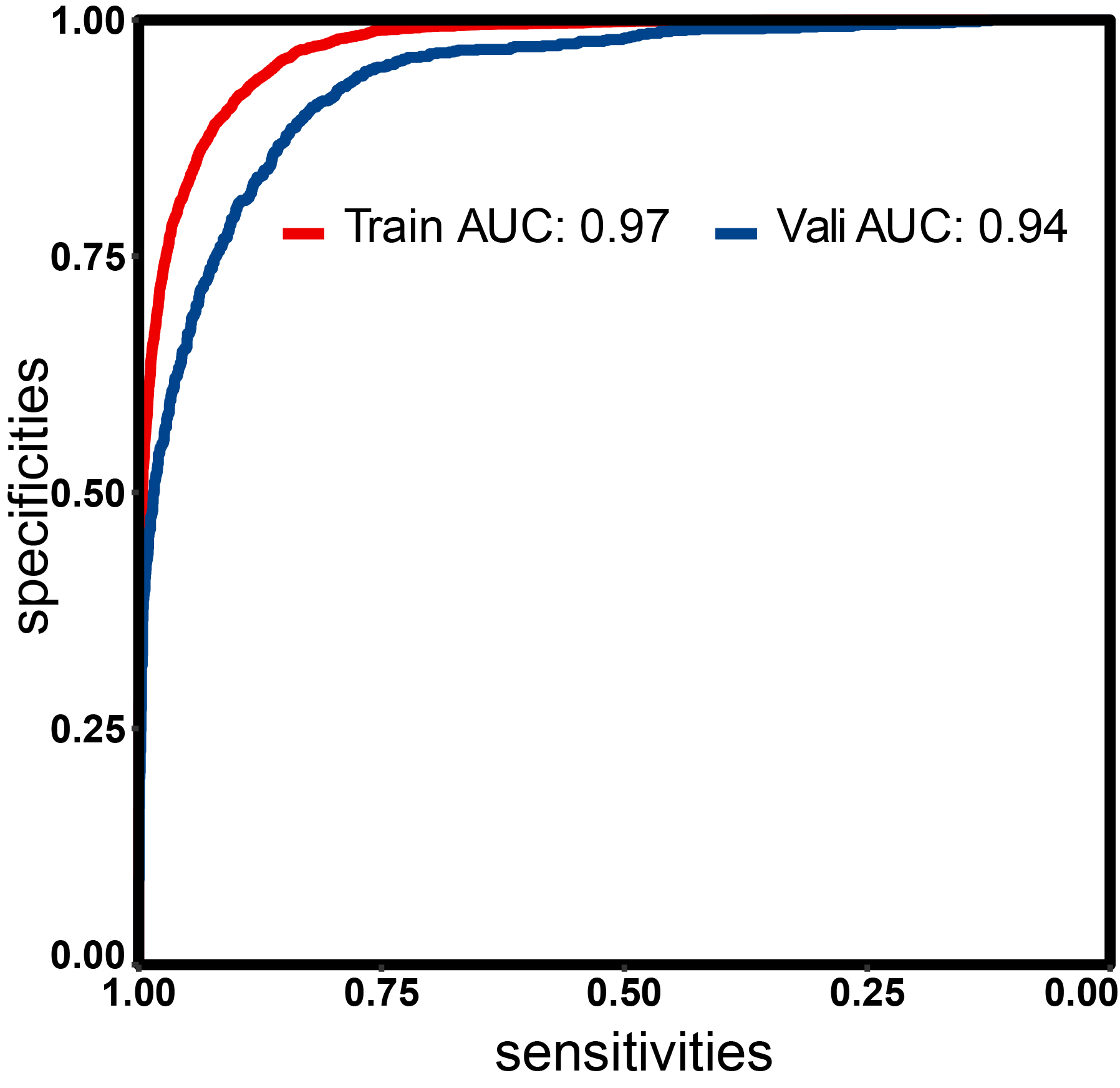


**Supplementary Figure 9.** Training set (GSE198052) and validation set (GSE116256) AUC results.
